# Synthesis and Investigation of Antibacterial Activity of Thin Films Based on TiO_2_-Ag and SiO_2_-Ag with Potential Applications in Medical Environment

**DOI:** 10.3390/nano12060902

**Published:** 2022-03-09

**Authors:** Cristina-Ș. Adochițe, Cătălin Vițelaru, Anca C. Parau, Adrian E. Kiss, Iulian Pană, Alina Vlădescu, Sarah Costinaș, Marius Moga, Radu Muntean, Mihaela Badea, Mihaela Idomir

**Affiliations:** 1 Faculty of Medicine, Transilvania University of Brasov, B-dul Eroilor nr 29, 500036 Brasov, Romania; cristina.adochite@unitbv.ro (C.-Ș.A.); sarah.costinas@gmail.com (S.C.); moga.og@gmail.com (M.M.); mihaela.idomir@unitbv.ro (M.I.); 2 National Institute of Research and Development for Optoelectronics-INOE 2000, 077125 Magurele, Romania; anca.parau@inoe.ro (A.C.P.); kadremil@yahoo.com (A.E.K.); iulian.pana@inoe.ro (I.P.); alinava@inoe.ro (A.V.); 3 Physical Materials Science and Composite Materials Centre, Research School of Chemistry & Applied Biomedical Sciences, National Research Tomsk Polytechnic University, Lenin Avenue 43, 634050 Tomsk, Russia; 4 Faculty of Civil Engineering, Transilvania University of Brasov, B-dul Eroilor nr 29, 500036 Brasov, Romania; radu.m@unitbv.ro

**Keywords:** antibacterial film, medical application, antibacterial properties, silver particle

## Abstract

Multiple antibiotic resistance has now become a major obstacle to the treatment of infectious diseases. In this context, the application of nanotechnology in medicine is a promising alternative for the prevention of infections with multidrug-resistant germs. The use of silver as a powerful antibacterial agent has attracted much interest. TiO_2_ and SiO_2_ thin films enhanced with Ag particles have been developed with the aim of maintaining the transparency of the polymer films. Antibacterial activity was evaluated for a Gram-negative species-*Escherichia coli*-in concentrations of 10^5^ and 10^4^ CFU/mL in different conditions-activation by UV irradiation, single layer and double layer. Increased antibacterial efficacy of TiO_2_-deposited foil was found for the tests that had been exposed to UV activation. In the case of bilayer tests, the efficiency was higher compared to those in a single layer, as the contact surface between the films and the bacterial suspension increased. Films can be used as a potential method to limit bacterial growth on hospital surfaces, such as telephone screens and medical equipment, given their optimized characteristics and proven antibacterial efficacy.

## 1. Introduction

Bacterial and viral infections, as well as other public health problems considered incurable diseases, have been effectively managed in recent decades due to remarkable advances in medicine. However, poor living conditions and a lack of knowledge about hygiene are contributing to high morbidity in many parts of the world, especially in less developed countries. Infectious pathology can also aggravate the evolution of other diseases and increases the mortality rate [1,2]. In recent decades, antibiotic resistance of many pathogens has been constantly increasing, becoming a major threat to global public health [3].

Since 2014, World Health Organization (WHO) reports have drawn attention to this serious threat to public health and recommended harmonizing and streamlining public health strategies and finding new alternatives to infection prevention and therapy. Data collected by the European Antimicrobial Resistance Surveillance Network (EARS-Net) in 2011–2019 indicated high levels of resistance to several classes of antimicrobials, with the highest percentages in Southern and Eastern Europe, including Romania [4,5]. Nosocomial infections are a serious problem facing hospitals around the world, with a significant medical, social and economic impact. Surveillance and control of healthcare-associated infections involves the development of appropriate strategies and the implementation of determined measures. Sources of infection can be exogenous, such as air, medical equipment and medical staff’s hands, or endogenous, such as normal skin bacterial flora [6]. The hands of medical staff play an important role in transmitting nosocomial infections. Therefore, contaminated mobile phones, which are used in various hospital wards, laboratories or halls, may play a role in the spread of microorganisms in medical units [7,8]. Unfortunately, the phones of medical staff are rarely disinfected and the transmission of microorganisms, including multidrug-resistant strains, is favored [9,10]. Gram-negative bacterial species that may be often agents of hospital infections are *Escherichia coli, Klebsiella pneumoniae, Pseudomonas aeruginosa* and *Acinetobacter baumannii*. Among the Gram-positive bacteria, methicillin-resistant *Staphylococus aureus* (MRSA) and Enterococcus species are more frequently implicated [5,11,12]. Antimicrobial surfaces have the potential to play a key role in infection management in healthcare institutions by decreasing cross-contamination and managing the surface bacterial load [13]. Given the above, it is possible to understand the need for methods to limit antibacterial infections by minimizing bacterial growth in hospital conditions. Thus, several researchers have focused on developing new antibacterial materials for screen phones, medical devices or medical equipment.

Most current research has focused on developing metal oxide nanoparticles for application as novel antibacterial materials by integrating scientific techniques with the natural antibacterial activity of inorganic metal oxides [14,15]. Nanomaterials of the next generation that can be activated by an external stimulus to gain antibacterial capabilities are an exciting step forward in the development of antimicrobial alternatives. Often, the nanomaterial’s antibacterial activity is also to blame for the related adverse effects, such as dissolved ions [16]. Nanomaterials that have been stimulated can, however, stay “dormant” until they are selectively “turned on,” lowering the risk of harmful side effects on human cells or helpful microorganisms [17]. Current stimuli-triggered antimicrobial nanomaterials use light and magnetism as their main stimuli, with various methods of action in each case. Photocatalytic and photothermal nanomaterials are driven by energy from specific wavelengths of light to produce reactive oxygen species (ROS) and localized temperature rises, which have been shown to be efficient against harmful bacteria and fungi, respectively. Magnetic hyperthermia and magnetophysical nanomaterials respond to magnetic fields by increasing the localized temperature and rupturing, respectively, to kill microorganisms [17]. Metal oxides are a viable alternative to the conventional antibacterial techniques since most have antibacterial properties. Ag_2_O, ZnO, SiO_2_, TiO_2_, CuO, MgO and CaO are some of the metal oxides that are often used as antibacterial materials [18,19]. Because of their high catalytic activity, great chemical and thermal stability, low toxicity and inexpensive cost, titanium dioxide (TiO_2_) nanoparticles are considered one of the most suited materials. Many benefits have been attributed to TiO_2_, including its high quantum efficiency, low cost, biocompatibility and great optical and chemical stability [20,21]. Deposition on TiO_2_ of noble metals such as silver and gold is one of the most effective ways to limit the possibility of electron–hole recombination in photocatalytic processes. Silver is frequently used with TiO_2_ because of its diverse photocatalytic and antibacterial characteristics [22,23]. Due to the carriage of silver-resistance bacterial genes by plasmids, the presence of silver-coated surfaces could contribute to the selection and propagation of antimicrobial-resistance genes [24]. Silica nanoparticles have proven to be a viable option for a variety of medicinal applications, including cancer and antibacterial treatments. Given the mounting threat of antimicrobial resistance, silica nanoparticles’ adaptability is particularly helpful for antimicrobial therapies, including biofilm treatment. The window for the development of antimicrobial resistance is quite narrow because these nanoparticles can attack pathogens through multiple modes, including physical damage to cell membranes, ROS production and endo-lysosomal burden, in addition to the antimicrobial activity induced by the cargo itself [25]. By attaching to the bacterial cell wall and/or releasing metallic ions, metal-based nanoparticles can disturb the cell membrane potential and integrity [26]. Because nanoparticles have a positive charge and cellular components have a negative charge, they interact at the surface through electrostatic communication. These interactions harm bacterial proteins by disrupting the membrane and increasing oxidative stress [27].

The aim of this study is to evaluate the antibacterial activity of polyurethane self-adhesive foils coated by TiO_2_ and SiO_2_ thin films enhanced with silver particles, in various conditions such as UV exposure and film doubling, to improve antimicrobial efficiency, as a potential material to protect screens used in medical environments.

## 2. Materials and Methods

### 2.1. Synthesis and Characterization of Films

The silver-containing thin film was obtained by the magnetron sputtering technique [24], in so-called confocal configuration. This configuration employs 3 targets located at equal distance from substrate holder position, in such a way that uniform deposition on a 9 cm diameter of the substrate is obtained. Each of the materials can be deposited individually, resulting in multilayer structures [27,28], or simultaneously-depositing 2 or 3 materials—resulting in a mixed uniform composition [29].

Both SiO_2_ and TiO_2_ targets operated in Radio Frequency (RF) mode at 50 W, having a constant power for the deposition of the dielectric matrix. Ag was obtained by sputtering the silver target (purity of 99.99%) in high-power impulse magnetron sputtering mode, HiPIMS [30], allowing us to obtain higher ion fluxes and also good control of the deposition rate through the temporal characteristics of the pulse. The pulse voltage was set at 650 V, with a peak current of 1.5 A, a pulse duration of 50 μs and the repetition frequency of 1 Hz. This allowed us to obtain a deposition rate that was typically 10 times smaller than that of the oxide. The substrates were made of thin self-adhesive polyurethane foils. The foils were cleaned with isopropyl alcohol before placing them into the vacuum chamber, and then additionally cleaned by RF sputtering at 50 V self-bias for 15 min. The deposition was performed in argon at 6 mTorr pressure, and the deposition rates were chosen so that the oxides remained the main material and the silver was merely a dopant. The typical deposition time was 30 min, resulting in film thicknesses ranging in the 30 to 35 nm interval.

UV-spectrophotometry was performed using a Jasco V-670 UV-Vis/NIR Spectrophotometer (Jasco, Tokyo, Japan). A SEM-Hitachi Tabletop Microscope (TM3030-PLUS, Tokyo, Japan) system equipped with an energy-dispersive X-ray spectrometer (EDS, QUANTAX 70, Bruker, Billerica, MA, USA) was used for the investigation of the composition of thin films.

The adhesion between the coated surface and uncoated substrate was determined by the “tape test”, performed according to the protocol described in the ASTM D3359-17 standard [31]. The test was carried out with an Elcometer 107 Cross Hatch Adhesion Tester kit (Ulmer, Aalen, Germany). The coating adhesion was evaluated in terms of area removed by analyzing the lattice pattern indentation and was classified in terms of percentages (from highest to weakest): 5B: 0%; 4B: ≤5% 3B: 5% ÷ 15%; 2B: 15% ÷ 35%; 1B: 35% ÷ 65%; 0B: ≥65%. A more detailed presentation of the procedure is available in reference [31].

### 2.2. Bacterial Culture

The antibacterial performance of films was assessed using *Escherichia coli* strains (ATCC 25922) in accordance with ISO 22196:2011 [32], although with various revisions based on the type of films tested, as explained below. A stock suspension of *Escherichia coli* of 0.5 McFarland equivalent to 1.5 × 10^8^ CFU/mL was prepared using a densitometer. Serial dilutions of 10^5^ and 10^4^ CFU/mL were made from this stock suspension. The films were inoculated with the bacterial suspension (V = 113 μL) for 30 min. at room temperature, after which they were washed in 3 mL of NaCl 0.9%. A volume of 100 μL was taken from the washing liquid and inoculated onto blood agar. Petri dishes with blood agar were analyzed after 24 h and also 48 h of incubation in a thermostat.

### 2.3. Antibacterial Culture

Comparative tests were performed in which the samples were exposed to UV radiation from a source placed at 30 and 60 cm, respectively, in order to activate the deposition under UV stimuli-taking into account the fact that some materials deposited on the samples had photocatalytic properties. At the same time, tests were performed to evaluate the antibacterial activity of only one sample, but also of two overlapping samples facing each other with the deposited side (sandwich) for the same volume of bacterial suspension. 

After 24 h and, respectively, 48 h of incubation in the thermostat of the Petri dishes, the number of *Escherichia coli* colonies was quantified using an automatic analyzer (InterScience Scan 300-Soft Scan InterScience, Saint Nom la Brétèche, France).

The results were analyzed and the average and standard deviations between the two experiments were calculated. The antibacterial efficiency of the tested samples was calculated using Equation (1) [33]:

(1) $$\mathrm{Antimicrobial}\;\mathrm{activity}\;\left(\%\right)=\frac{N_{c}-N_{s}}{N_{c}}\times 100$$

where:

N_c_ represents the number of colonies on the control samples;

N_s_ represents the number of colonies on the tested samples.

## 3. Results

### 3.1. Characteristics of the Films

**Figure 1:**
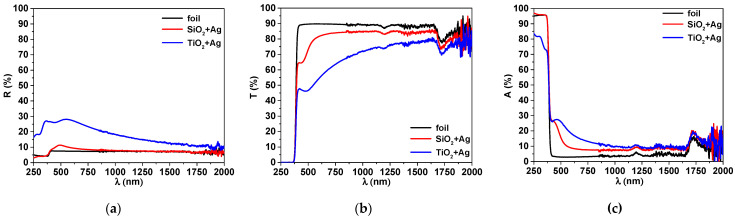
Spectrophotometric curves, reflectivity (**a**), transmittance (**b**) and absorbance (**c**) of the uncoated foil and the foils coated with SiO_2_ + Ag, TiO_2_ + Ag.

The optical properties of the resulting structure were investigated in this study through UV–Vis-NIR spectroscopy, to assess the changes induced by the thin films deposited on the transparent polymer foil (Figure 1). Maintaining high transparency is an important feature, enabling the use of such foils as protective covers for touchscreens or other surfaces that require see-through characteristics.

From the transmission and reflection spectrum presented in Figure 1, it may be observed that the transparency decreases when Ag particles are added into the oxide matrix. The transparency is significantly lower for the TiO_2_ + Ag layer, the decrease being associated both with higher reflectivity, up to 20%, and slightly higher and broader absorption in the visible range. An absorption peak around 450–500 nm is present for both silver-containing coatings, as can be seen from the absorption spectra in Figure 1. This peak is specific to the absorption on silver nanoparticles, being associated with the surface plasmon resonance phenomena [34]. It is evident that the peak position depends on the matrix, indicating that the size and density of the Ag nanoparticles are different, depending on the matrix they are embedded in [35] The presence of this peak confirms that the Ag nanoparticles are finely dispersed in the oxide matrix. Comparing both layers, the one with the SiO_2_ matrix has the best transparency, the one containing TiO_2_, on the other hand, being less transparent, most probably due to an insufficient amount of oxygen and a corresponding sub-stoichiometric composition [36].

**Figure 2:**
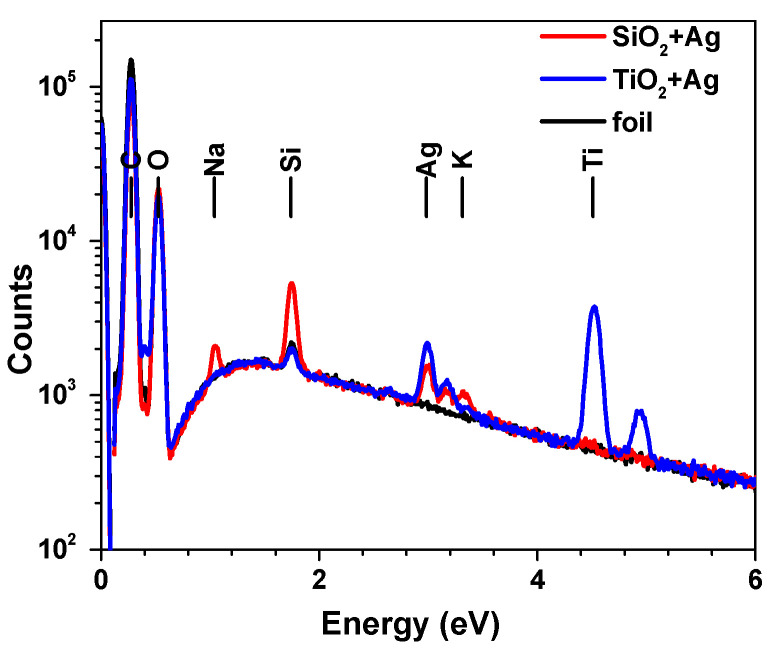
EDS spectra and element distribution of the polymer foils and the foils deposited with TiO_2_ + Ag films, SiO_2_ + Ag; all films have a total thickness of ~33 nm.

The film composition was assessed by EDS (Figure 2). A measurable amount of Ag was detected for both types of thin films. The ratio between Ag and Si is around 23%, whereas the ratio between Ag and Ti is around 28%, showing a slightly higher content of Ag in the TiO_2_ matrix. When comparing the nominal ratios of Ag embedded in the two dielectrics, the difference is even higher, with 0.13% from the total in SiO_2_ compared with 0.23% in TiO_2_.

**Figure 3:**
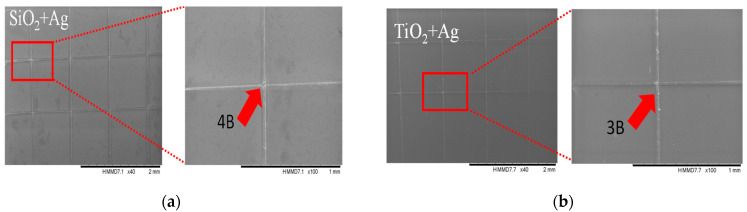
Illustrative SEM images of thin films after adhesion tests (red-colored arrow indicates the delamination/detached area) for SiO_2_ + Ag (**a**) and TiO_2_ + Ag (**b**).

In Figure 3, the SEM images of the coatings after performing the adhesion assays by the “tape test” are presented. Similar examinations were also performed by other researchers [37,38], who investigated the adhesion between hydroxyapatite-based thin films deposited on titanium substrates used for medical applications. The SEM results of the adhesion tests revealed that the Ag-doped SiO_2_ thin films have strong adhesion to the uncoated substrate classified according to the ASTM D 3359-17 standard at the top of the category, identified as 4B, indicating only small detachments of film in line with indentations or at cut intersections. By adding the Ag into TiO_2_ thin films, we observed a much more pronounced delamination/detached area, in the range of 5 ÷ 15% according to the ASTM D 3359-17 standard, and thus the adhesion falls into the 3B category of the standard.

### 3.2. Antibacterial Activity

Partial results of this work were published and described the synthesis of TiO_2_-Ag and SiO_2_-Ag films deposited on polyurethane self-adhesive substrates by a combined sputtering technique, namely RF sputtering for the oxide and HiPIMS for Ag, and also the complete structural characterization and evaluation of the antimicrobial activity at 24 and 48 h for the films in contact with *Escherichia coli* suspensions of concentrations 10^5^ CFU/mL and 10^4^ CFU/mL [39]. In addition, in this paper, the advantages and the novelty of the study are included, as well as the extension of tests in different conditions, which are described below.

An experiment for evaluating the antibacterial activity was conducted using unmodified foil, considered a reference sample. The antibacterial activity was evaluated according to the incubation period of the Petri dishes-24 h and 48 h, respectively-and according to the concentration of the *Escherichia coli* suspension-10^4^ and 10^5^ CFU/mL. The antibacterial activity is concentration-dependent as well and it is unaffected by the bacteria’s acquisition of antibiotic resistance. A recent study performed by Ayala-Núñez et al. [40] demonstrated that Ag particles have dose-dependent antibiotic activity against MRSA and non-MRSA, and that, at concentrations above 1.35∙10^−3^ μg∙mL^−1^, both MRSA and non-MRSA are suppressed at inoculum concentration 10^5^ CFU/mL [11]. 

The cell wall of *Escherichia coli* bacteria contains a negative charge, which aids the electrostatic contact between Ag particles and bacteria [40]. The generated Ag^+^ ions cross the cell membrane and bind with the thiol groups of proteins, inhibiting DNA replication and potentially killing the bacteria [17]. 

**Figure 4:**
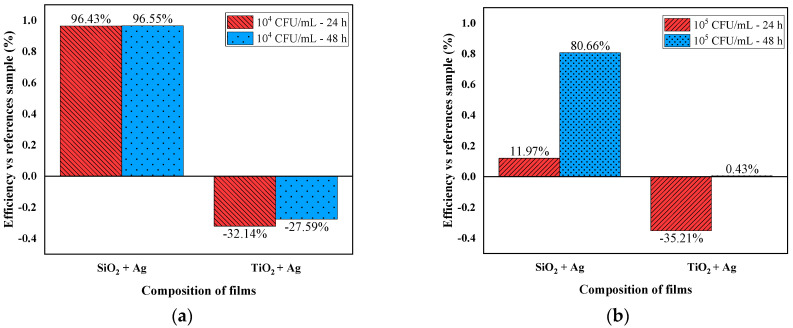
Antibacterial efficacy depending on the type of film and on the incubation time-24 h and 48 h-and the applied *Escherichia coli* inoculum concentration: (**a**) 10^5^ CFU/mL and (**b**) 10^4^ CFU/mL.

Figure 4a reveals that samples coated by SiO_2_ + Ag at a concentration of 10^5^ CFU/mL have 11.97% efficiency after 24 h of incubation and 80.66% after 48 h of incubation, hence indicating poorer bacterial growth than the reference sample. The samples coated by TiO_2_ + Ag, under the investigated conditions, showed enhanced bacterial growth as compared to the reference sample (polymer foil sample without thin film).

Figure 4b shows a percentage of 96.43% for the antimicrobial efficiency of the sample coated by SiO_2_ + Ag, at the concentration of 10^5^ CFU/mL, after 24 h and 96.55% after 48 h of incubation, so bacterial growth was much poorer at both incubation intervals than the reference sample. As a result, increased antibacterial potential was obtained at the concentration of 10^4^ CFU/mL as compared to the tests performed at the concentration of 10^5^ CFU/mL for the samples coated by SiO_2_ + Ag. For samples coated by TiO_2_ + Ag, the bacterial proliferation may have occurred due to a lack of UV radiation stimuli, given that ROS species interact with the bacterial cell membrane. The ROS are thought to first interact with the bacterial membrane, causing oxidative damage and breaking the cell wall, exposing the bacteria’s internal compartment to the external environment. This action causes the uncontrolled flow of components into and out of the cell, eventually leading to cell death [41,42].

Antibacterial nanomaterials can be activated by various factors and methods, more often by light or magnetism. Photocatalytic and photothermal nanomaterials are stimulated by energy from specific wavelengths of light to produce reactive oxygen species (ROS) and localized temperature rises, which has been shown to be efficient against harmful bacteria and fungi, respectively [17].

**Figure 5:**
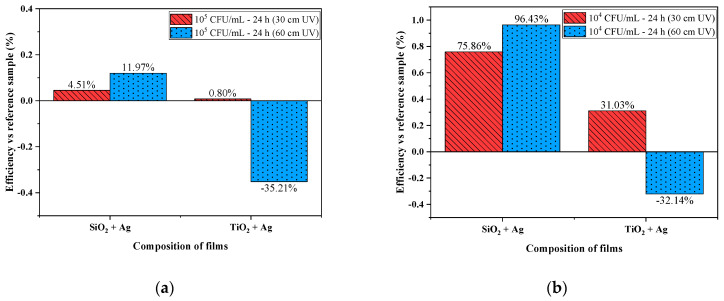
Antibacterial efficacy of UV-treated coatings (30 and 60 cm) for activation at different concentrations of *Escherichia coli* inoculum: 10^5^ CFU/mL (**a**) and 10^4^ CFU/mL (**b**).

Considering the fact that TiO_2_ is a very good photocatalyst in UV radiation [43], the tests were performed to activate the materials at different distances from the UVC lamp in the micro-biological hood-30 and 60 cm, respectively (Figure 5).

Experiments for evaluating the antibacterial activity were conducted using an unmodified sample (polymer foil sample without thin film) irradiated with UV at 30 and 60 cm, considered a reference sample. The Petri dishes were evaluated after 24 h of incubation in thermostat.

In the case of tests performed at concentrations of 10^5^ CFU/mL at 30 cm distance of irradiation (Figure 5a), the antibacterial efficiency compared to the reference sample was reduced for the sample coated by SiO_2_ + Ag (11.97% antibacterial efficiency), and for the sample with TiO_2_ + Ag, a lower degree of bacterial growth was recorded (0.80% antibacterial efficiency). A lower degree of bacterial growth was observed when activated at 30 cm, corresponding to the samples with SiO_2_ + Ag (4.51% antibacterial efficiency). For tests performed at 60 cm from UV radiation, for the SiO_2_ + Ag sample, high percentages of antibacterial efficiency were maintained compared to the TiO_2_ + Ag sample. A possible explanation for this result could be due to the fact that silica nanoparticles change their chemical composition and reorganize their structure following UV irradiation, which leads to a correlation with the decrease in antibacterial efficiency at a concentration of 10^5^ CFU/mL. Another possible explication would be a photorefractive effect caused by the production of hydroxyl groups (SiOH), which causes alterations in the SiO_2_ material’s optical index of refraction [44].

Figure 5b shows high percentages of antibacterial efficiency for the SiO_2_ + Ag material at a concentration of 10^4^ CFU/mL. For the sample coated by TiO_2_ + Ag, a percentage of 31.03% antibacterial efficiency was determined for UV activation at a distance of 30 cm, and at a distance of 60 cm, a percentage of −32.14% was registered (higher bacterial growth compared to the reference sample). In this system, silver might help to build Schottky barriers at the Ag/TiO_2_ interfacial contact area, which would reduce the electron–hole recombination in the photocatalytic process [45,46]. The same trend was registered for the plates incubated for 24 h; the layer with SiO_2_ + Ag and irradiated with UV stimuli at a 60 cm distance had the highest efficiency of the tested materials-96.43% antibacterial efficiency—while, with the concentration of 10^4^ CFU/mL, the percentages were higher than those for the concentration of 10^5^ CFU/mL-11.97% antibacterial efficiency for tests performed under these conditions. The antibacterial effects of photocatalytic nanomaterials are based on the generated ^•^O_2_^−^, ^•^OH radicals and H_2_O_2_. Additionally, photocatalysis with metal nanomaterials has been used to demonstrate the formation of singlet oxygen (1O_2_), a powerful oxidation reagent [47]. Although the antibacterial action of these ROS has not been definitively identified, it is hypothesized that a variety of mechanisms are involved [48].

**Figure 6:**
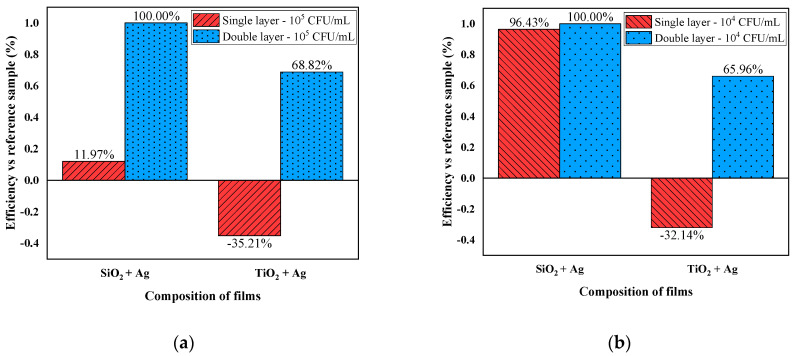
Antibacterial efficacy of films for concentrations other than *Escherichia coli*-10^5^ CFU/mL (**a**) and 10^4^ CFU/mL (**b**)-for single-layer experiments and double-layer tests (sandwich).

To demonstrate the efficacy of the deposited thin films, tests were performed that analyzed the antibacterial efficacy for a single sample compared to the tests in which two samples were used facing each other, with bacterial inoculum placed between the two faces deposited with thin films. The purpose of this test was to record the increased variability in the number of bacterial colonies for experiments performed using a single deposited layer, which leads to uneven coverage of the surface with the bacterial inoculum. To avoid this factor, a second foil was used on top of the first one, to increase contact with the bacterial suspension (Figure 6).

Notably, Ag particles’ adhesion to cell membranes and consequent lipid bilayer changes result in enhanced membrane permeability, damage and cell death, a powerful antibacterial action that appears to be more pronounced when smaller nanoparticles are utilized [49].

Tests performed using a single layer of SiO_2_ + Ag showed a percentage of 11.97% for antibacterial efficiency, for the concentration of 10^5^ CFU/mL of *Escherichia coli* suspension, and for the concentration of 10^4^ CFU/mL, efficiency of 96.43% was observed. Compared to these results, the double layers registered a percentage of 100% antibacterial efficiency. A significant difference was also indicated in the case of TiO_2_ + Ag layers, where, in the case of the single layer, there was a proliferative effect in contact with the material, while, in the case of doubling the contact surface, the percentages of antibacterial efficiency were 68.82% (*Escherichia coli* 10^5^ CFU/mL) and 65.96% (*Escherichia coli* 10^4^ CFU/mL). Because both types of foils dispersed the Ag particles evenly, it was observed for both concentrations of *Escherichia coli* that the efficiencies were higher in the case of the bilayers compared to the tests where a single layer was used. A study performed in 2020 by Pal, Tak and Song [50] showed that the surface area to volume ratio of silver nanoparticles, as well as the crystallographic surface structures, are critical determinants in silver nanoparticles’ antibacterial efficacy.

The positive charge induces electrostatic attraction between silver nanoparticles and the microorganisms’ negatively charged cell membranes, allowing silver nanoparticles to adhere to cell membranes more easily. When such interactions occur, morphological changes such as cytoplasm atrophy and membrane separation occur, eventually leading to cell wall rupture [51]. The cell membrane of *Escherichia coli* cells becomes entirely broken after a few minutes of interaction with silver nanoparticles, according to transmission electron microscopy [52]. As demonstrated by TEM, the cell wall becomes circumferential, and many electron-dense pits develop at silver nanoparticle-induced damage areas [11,53].

## 4. Conclusions

Thin films consisting of an oxide matrix with silver nanoparticles were obtained on polymer self-adhesive foils, using magnetron sputtering. The antibacterial activity on *Escherichia coli* has been shown to be more effective for SiO_2_ + Ag films. A potential way to improve the antibacterial effects of the material with TiO_2_ + Ag is to activate it with UV radiation. It was shown that, for the foil deposited with TiO_2_ + Ag under conditions of UV irradiation at a distance of 30 cm from the films, the antibacterial efficiency was almost 30 times higher for the concentration of *Escherichia coli* of 10^4^ CFU/mL compared to irradiation at 60 cm from samples. As a result, it was observed that the demonstrated photocatalytic activity of TiO_2_ may increase the antibacterial efficacy under the described experimental conditions. Moreover, using a setup that doubles the interaction surface by placing the inoculum between two facing active surfaces, it was shown that the adhesion to the bacterial suspension is increased. This is therefore an alternative way to evaluate the antimicrobial activity. Films can be used as a potential method to limit bacterial growth on hospital surfaces, such as telephone screens and medical equipment, given their optimized characteristics and proven antibacterial efficacy [54].

## Data Availability

Not applicable.
